# Primary Metabolism in Fresh Fruits During Storage

**DOI:** 10.3389/fpls.2020.00080

**Published:** 2020-02-19

**Authors:** Stefano Brizzolara, George A. Manganaris, Vasileios Fotopoulos, Christopher B. Watkins, Pietro Tonutti

**Affiliations:** ^1^ Institute of Life Sciences, Scuola Superiore Sant'Anna, Pisa, Italy; ^2^ Department of Agricultural Sciences, Biotechnology & Food Science, Cyprus University of Technology, Lemesos, Cyprus; ^3^ School of Integrative Plant Science, College of Agriculture and Life Sciences, Cornell University, Ithaca, NY, United States

**Keywords:** low temperature, controlled atmospheres, modified atmospheres, hypoxia, ethylene, 1-methylcyclopropene, fruit composition, post-harvest

## Abstract

The extension of commercial life and the reduction of postharvest losses of perishable fruits is mainly based on storage at low temperatures alone or in combination with modified atmospheres (MAs) and controlled atmospheres (CAs), directed primarily at reducing their overall metabolism thus delaying ripening and senescence. Fruits react to postharvest conditions with desirable changes if appropriate protocols are applied, but otherwise can develop negative and unacceptable traits due to the onset of physiological disorders. Extended cold storage periods and/or inappropriate temperatures can result in development of chilling injuries (CIs). The etiology, incidence, and severity of such symptoms vary even within cultivars of the same species, indicating the genotype significance. Carbohydrates and amino acids have protective/regulating roles in CI development. MA/CA storage protocols involve storage under hypoxic conditions and high carbon dioxide concentrations that can maximize quality over extended storage periods but are also affected by the cultivar, exposure time, and storage temperatures. Pyruvate metabolism is highly reactive to changes in oxygen concentration and is greatly affected by the shift from aerobic to anaerobic metabolism. Ethylene-induced changes in fruits can also have deleterious effects under cold storage and MA/CA conditions, affecting susceptibility to chilling and carbon dioxide injuries. The availability of the inhibitor of ethylene perception 1-methylcyclopropene (1-MCP) has not only resulted in development of a new technology but has also been used to increase understanding of the role of ethylene in ripening of both non-climacteric and climacteric fruits. Temperature, MA/CA, and 1-MCP alter fruit physiology and biochemistry, resulting in compositional changes in carbon- and nitrogen-related metabolisms and compounds. Successful application of these storage technologies to fruits must consider their effects on the metabolism of carbohydrates, organic acids, amino acids and lipids.

## Introduction

Harvested fruits are treated with a range of postharvest technologies to mantain quality by delaying ripening and senescence, preventing the incidence of physiological and pathological disorders, and avoiding water loss and physical damage. The main pillar of fruit storage is the use of refrigeration. Temperate fruit crops are commonly stored at temperatures close to freezing (0–1°C), while those of tropical or subtropical origin must be kept at higher storage temperatures (7–15°C) to avoid losses due to the development of chilling injury (CI) symptoms. These symptoms can be manifested as altered ripening behavior and external (peel) and/or internal (flesh) damages (browning, pitting, breakdown, discoloration), being more evident after subsequent removal from cold storage and maintenance at room temperarure. Appropriate storage temperatures can extend storage life by about 2–4 weeks for crops such as apricots, sweet cherries, and peaches, and up to several months for apples, pears, and kiwifruits.

Storage protocols that reduce oxygen and/or increase carbon dioxide concentrations in combination with low temperature are mainly used for fruits such as apples, pears, and kiwifruits. Similar conditions are also applied with modified atmosphere (MA) storage, usually applied as MA packaging (MAP) for minimally processed (fresh cut) products and to a lesser extent as whole fruits. For controlled atmosphere (CA) storage, the atmosphere composition is strictly monitored and adjusted in gas tight rooms by control systems, whereas in MA the changes in oxygen and carbon dioxide concentrations within the package are a function of factors such as the respiration rate of the produce as affected by cultivar, ripening stage, weight, and temperature in combination with packaging film characteristics. Optimum CA/MA storage regimes for different fruit types have been mainly developed empirically based on their quality after storage.

The main effects of low temperature and the CA/MA storage alone or in combination are associated with respiration, ethylene biosynthesis and its action, and other metabolic processes, thereby decreasing the rates of change that occur during post-harvest ripening, including color (chlorophylls, carotenoids, and flavonoids), texture (softening as a result of cell wall disassembly and reduced cell turgor), and flavor (taste and aroma as a result of starch degradation, sugar-acid metabolism, and synthesis of aromatic volatiles). These effects can apply regardless of whether the fruit is non-climacteric or climacteric, but for the latter fruit types are important for reduction of ethylene production. Ethylene has a key physiological role during the ripening process, a genetically regulated stage of development of climacteric fruits that is highly complex and coordinated by hormonal metabolism. In addition to physical methods, a chemical antagonist of ethylene, 1-methylcyclopropene (1-MCP), is used on specific fruit types. 1-MCP effects vary depending on the species, cultivar, maturity and ripening stage, and factors such as 1-MCP concentration, treatment duration and temperature, and post-treatment storage conditions. It is commercially applied to several commercially important fleshy fruits, such as apples and pears

Storage technologies have profound effects on primary metabolism with marked consequences on the composition and the overall flavor of the commodity and, hence, the commercial life and the consumer acceptance. The objective of this review is to provide a perspective of these effects on the metabolism of carbohydrates, organic acids, amino acids, and lipids in fruits.

## Low-Temperature Storage

Low temperature is the main method used to extend storage and market life of fruits. By reducing general metabolism, cold storage protocols delay ripening and senescence processes through the control of the respiration rate. Low temperature is effective in decreasing the catalytic activities of different enzymes, including those involved in the different steps of respiration. The changes induced by temperature in a biological system are measured by using the Q10 temperature coefficient. This coefficient indicates the rate of reactions as a result of a temperature increase of 10°C (Atkin and Tjoelker, 2003). For most of harvested fruit crops, within a temperature range of 5–25°C, Q10 values associated with respiration are, in general, between 2.0 and 3.0. This indicates that when lowering storage temperature from 20 to 10°C the respiration is decreased by a factor of 2–3, with positive consequences in terms of shelf/commercial life of the produce. The optimum low-temperature and storage duration are highly variable based on the fruit type and are applied keeping in mind two principles: (1) low temperatures slow down general metabolism and reduce the rate of compositional changes; and (2) temperatures lower than a specific threshold and/or a prolonged cold storage induce physiological disorders. For the non-chilling sensitive fruit types, the storage temperatures are set just above their freezing point. Other types, e.g., tropical or sub-tropical fruits, must be stored at higher temperatures, typically between 7 and 15°C. Factors such as cooling rates and precooling techniques also play a key role in determining the success of cold storage (Valero and Serrano, 2010). As a general effect, low-temperature storage upregulates stress-responsive genes, blocks signal transduction of ethylene-related processes, and affects both primary and secondary metabolism (Yun et al., 2012; Lin et al., 2018). Changes at the level of secondary metabolism during low-temperature storage have been extensively studied, mostly focusing on how cell wall alterations affect abnormal ripening (Brummell et al., 2004). Despite the well-documented CI-induced activation of genes that are involved in different metabolic pathways (Gonzalez-Aguero et al., 2011), information about the effects of cold storage on fruit primary metabolism remains limited.

### Carbohydrate Metabolism

Carbohydrates influence the sensitivity of plant tissues and organs, including fruits, to low temperature. Besides being an important fruit component markedly impacting the overall flavor, soluble carbohydrates may have several beneficial effects in protecting plants against stresses, including cold storage regime, and the relationship between CIs and carbohydrate metabolism was investigated in an array of fruit types. Special focus has been addressed to commercially important fruits, such as apples, pears, kiwifruits, and bananas that, at physiological maturity, are characterized by high starch contents that are converted to sugars during storage. An emerging research area is the impact of carbohydrate composition and contents on the sensitivity of fruits to low temperatures, although results are inconsistent (Zhao H. et al., 2019). Furthermore, over the recent years, a number of studies reported the effects of applications of chemicals on CI development in fruits in relation to sugar metabolism and/or changes in carbohydrate content. Excellent examples of sugar metabolism during cold storage of two distinct fruit types (peach, mandarin), which are characterized by abnormal ripening after cold storage, are available.

Carbohydrate metabolism has been extensively studied in cold-stored peaches with differential responses of individual sugars, mainly associated with CI symptoms, evident as browning. Enhanced chilling tolerance in peaches has been associated with higher sucrose contents, resulting from the balance between its degradation and biosynthesis, which may contribute to membrane stability (Wang et al., 2013). Brizzolara et al. (2018) found that “Red Haven” peaches had higher contents of sucrose and sugar alcohols such as sorbitol and maltitol after cold storage and reduced susceptibility to CI compared with “Flaminia” and “Regina di Londa” fruits. Induction of chilling tolerance of nectarines stored at near freezing temperatures (−1.4 °C) has been associated to reduced activities of sucrose metabolism-associated enzymes that resulted in higher sucrose contents (Zhao H. et al., 2019). Sound peaches were characterized by higher activities of hexokinase, fructokinase, and energy metabolism-associated enzymes and higher content of sucrose and lower contents of fructose and glucose were associated with reduced CI induced by glycine betaine treatment (Wang L. et al., 2019).

In citrus, the carbohydrate changes occurring in the flavedo along with fruit maturation do not appear to be related to the chilling tolerance in cold stored “Fortune” mandarins (Holland et al., 1999). Pre-storage heat conditioning (3 days at 37°C) enhanced chilling tolerance of the fruits, favoring sucrose, but not hexose, accumulation. This effect was attributed to heat-induced increase in the activities of sucrose-synthesizing enzymes, such as SPS and SuSy (Holland et al., 2005). Heat treatment limited the decline in sucrose content of flavedo tissue during cold storage, evidenced by the substantially higher amounts of sugars in heat-conditioned compared with non-conditioned fruits (Holland et al., 2002). Heat conditioning led to loss of glucose, fructose, and starch in fruit kept subsequently at 2°C, suggesting that only sucrose is actively involved in the heat-induced chilling tolerance of citrus fruits.

Collectively, the role of sugar synthesis during cold storage on the incidence of CI symptoms should be evaluated on a species basis and also based on the nature and symptomology of each chilling related disorder. Comparative studies can be convincing, but it is not always certain that the variation among cultivars is smaller than variations between chilling-tolerant and chilling-sensitive cultivars; simple comparisons of two cultivar responses should be discouraged.

### Organic Acid Metabolism

There is increasing evidence that organic acids act not only as intermediates in carbon metabolism but also as key components in response to environmental stress factors (Lopez-Bucio et al., 2000). In most fruits, the organic acid pool decreases during ripening, and low temperature storage limits the rate of titratable acidity (TA) loss: this is imputed to the reduced metabolism, in particular respiration. In different kiwifruit cultivars, Cha et al. (2019) reported significantly lower TA values in fruit kept at 18°C compared to 10 and 5°C storage.

Considering the fate of specific organic acids during cold storage, a genotype-dependent behavior is present. Bustamante et al. (2016) showed that six different peach genotypes displayed a decrease in 2-oxoglutarate (2-OG) and succinate contents during refrigerated storage. Decreases of malic and quinic acid contents were observed in myrtle fruits stored at 2 and 10°C (Angioni et al., 2011; Mulas et al., 2013), indicating a possible shift of metabolic activity toward the biosynthesis of secondary metabolites such as anthocyanins. An increasing number of reports highlight the link between postharvest treatments performed prior to cold storage and organic acid metabolism. Zhou et al. (2019) demonstrated that UV-treated peaches stored at 1°C were characterized by a down-regulation of aconitase and NADP-malic enzyme activities and gene expression levels, but higher levels of citrate synthase and NAD-malate dehydrogenase, resulting in a reduced degradation of citric and malic acids. Interestingly, pre-cold storage hot air treatment (40°C, 48 h) in ponkan orange promoted citric acid degradation, attributed to regulation by ATP citrate lyase (ACL) and γ-aminobutyric acid (GABA) pathways (Gao et al., 2018).

### Amino Acid Metabolism

Amino acid metabolic responses of fruits to cold storage are species- and storage condition-specific. For example, Micro-Tom tomatoes stored at 4°C resulted in a rise in Glu, Gln, Asp, and Asn contents (Gonzalez et al., 2019). Cold storage also increased endogenous substrate proteolysis, azocaseinolytic activity, and free amino acid contents, but their specific roles have not been elucidated (Re et al., 2012). Similar approaches have been employed for other species such as kiwifruit, where cold storage resulted in increased Thr, Ile, and Val contents, but not of Gln and Asn (Salzano et al., 2019). In peaches, the beneficial effect of heat pre-conditioning to alleviate CI symptoms was linked with the modification of metabolites, including sugars, polyamines, and amino acid precursors of the phenylpropanoid pathway (Lauxmann et al., 2014). Bustamante et al. (2016) reported that cold storage of six peach cultivars at 0°C resulted in increased GABA, Asp, and Phe contents, and a genotype-dependent tolerance of peach cultivars to CIs was associated with higher amino acid contents (Brizzolara et al., 2018).

### Lipid Metabolism

Fatty acids are essential cell membrane components, constituting a selectively permeable barrier which represents an accessible fluid medium for lipophilic molecules/complexes and a matrix for enzymes that catalyze different metabolic reactions. Stress conditions alter membrane lipids, especially their level of unsaturation, altering membrane functioning, leading to ion leakage and cellular decompartmentalization (Marangoni et al., 1996). However, information about lipid metabolism of fruit during refrigerated storage is limited.

In peaches, the genotype affects the relative amounts of plastidic glycerolipid and triacylglyceride forms, possibly indicating their use as a source of energy during fruit senescence (Bustamante et al., 2018). Posphatidylethanolamine, phosphatidylcholine (PC), and digalactosyldiacylglycerol (DGDG) contents are possible markers of cold tolerance, given the important role played by the membranes in the development of CI symptoms. Wang Y. et al. (2019) found increased DGDG contents in blueberries after 30 and 60 days of storage at 0°C. Other lipids such as phosphatidic acid (PA) are accumulated in pineapple fruit during blackheart development at 10°C (Zhou et al., 2014). Accumulation of PA could be linked with previously observed increased activity of phospholipase D (PLD) in cold-stored fruits, as PLD hydrolyzes structural phospholipids such as PC to PA and a free-head group such as soluble choline (Wang, 1999). Sheng et al. (2016) and Shi et al. (2018) also measured increased PLD enzyme activities and transcript levels in cold-stored pears. Similarly, chilling injured “Honeycrisp” apples with soggy breakdown had elevated contents of glycerol and TAGs (Leisso et al., 2015).

## CA and MA Storage

An extensive amount of information is available regarding the responses of different fruit types to reduced oxygen and elevated carbon dioxide concentrations (Yahia, 2009; Gross et al., 2016; Thompson et al., 2018). At commercial level, CA storage is widely applied to extend storage potential of commercially important commodities such as apples, pears, and kiwifruits.

For apples, traditional CA storage regimes (oxygen concentrations above 1 kPa) is being replaced by use of ultra-low oxygen (ULO) concentrations (<1 kPa). Dynamic CA (DCA) allows use of much lower oxygen concentrations. Measurement of physiological/biochemical responses of fruit to low oxygen by chlorophyll fluorescence (Prange et al., 2005), ethanol production (Gasser et al., 2008) and the respiratory quotient (RQ) (Bessemans et al., 2016) is allowing storage closer to the anaerobic compensation point (ACP).

The effects of low-oxygen storage depend on the intensity and duration of the applied stress on fruit metabolism. The fruit type, cultivar, maturity and ripening stage, and pre-harvest conditions are also important factors affecting behavior of harvested fruits. Most of the fundamental information available concerning the primary metabolic changes in CA-stored fruits (mainly apples and, to a lesser extent, pears) derives from studies where both oxygen and carbon dioxide concentrations were altered. Therefore, it is not always easy to discriminate between the effects of the two conditions, especially where synergistic or additive effects occur, e.g. the responses of specific components and reactions of the tricarboxylic acid-cycle, and the fermentation products (acetaldehyde, ethanol, and ethyl acetate) (Kanellis et al., 2009).

### Carbohydrate Metabolism

A major effect of low oxygen concentrations in fruit responses is the switch from the aerobic to anaerobic metabolism to compensate for energy deficits. Severe oxygen deficiency reduces mitochondrial respiration resulting in limited ATP availability for energy-demanding processes. Fruit metabolism responds to this energy crisis by increasing substrate level ATP production through different processes, including the catabolism of soluble sugars and, in some species, of starch. For example apples, during the advanced developmental stages, accumulate starch that decreases during CA storage (Gorin et al., 1978). In rice seed hypoxia induces the up-regulation of β-*amylase* genes that are activated to satisfy the increased carbon demand by glycolysis (Planchet et al., 2017). β*-Amylase* is also induced by low oxygen in apples (Cukrov et al., 2016). Futile cycles involving sucrose/hexose interconversion are considered the main mechanisms producing the specific sugar types in fruit tissue in normoxia (Nguyen-Quoc and Foyer, 2001). Low oxygen concentrations affect sugar-metabolism-related enzyme activities and gene expression in fruits. However, contents and the fate of simple sugars (glucose, fructose) and sucrose in fruit under CA conditions are still not yet fully clarified. Different responses may be a function of different oxygen and carbon dioxide concentrations, temperatures, and storage durations as well as effect of genotype. The decline of sucrose contents after harvest is slower in CAs than in air for apples (Zhu et al., 2013) and peaches (Lara et al., 2011), and associated with lower invertase but higher SuSy activities (Zeng et al., 1999; Geigenberger, 2003). *SuSy* gene expression is highly induced in apples under hypoxic conditions, as also observed in the model species *Arabidopsis* (Mustroph et al., 2010), representing a key responsive gene to hypoxia in fruit tissues (Cukrov et al., 2016). *SuSy* induction has been related to the activation of alternative pathways that use inorganic pyrophosphate (PPi) instead of ATP phosphorylation reactions to compensate for severe ATP deficiency. In apples kept from 0.4 to 3.0 kPa oxygen, fructose and glucose do not show significant changes within 30–60 days of storage, probably due to similar rates of consumption and synthesis (Bekele et al., 2016; Cukrov et al., 2016). To meet the energy demand under low oxygen concentrations, an increase in carbohydrate flux through glycolysis occurs with the conversion of glucose-6-phosphate to pyruvate (Pasteur effect). The oxygen concentrations activating the responses leading to the Pasteur effect vary depending on fruit type and the ACP. The conversion of fructose-6-phosphate to fructose-1,6-biphosphate is catalyzed by phospho-fructo kinase (PFK) that is rapidly induced under hypoxia in apples. In “Granny Smith” apples, PFK is highly responsive to subtle changes in oxygen concentration (Cukrov et al., 2016), and, similarly to SuSy, uses PPi instead of ATP to save energy (Bailey-Serres et al., 2012). PFK most likely represents a key element for re-setting carbon metabolism under oxygen deficiency through an induction of the glycolytic pathway in rice (Mustroph et al., 2013). Both PFK and pyruvate kinase (PK) are highly and rapidly responsive to subtle changes of oxygen concentrations in apple tissues (Brizzolara et al., 2019). NADP^+^ and NAD^+^ must be regenerated to maintain the glycolytic flux and the concentration of pyruvate, which cannot be used in Krebs cycle in the absence of oxygen, must remain low (Bailey-Serres et al., 2012; António et al., 2016). To decrease its concentration and further produce ATP, pyruvate is redirected toward the fermentation pathways that produce lactate, acetaldehyde and ethanol with, at the same time, the maintenance of the redox balance in the cell by the formation of NAD^+^.

Ethanol production/accumulation occurs in apples stored under both static and DCA (Saquet and Streif, 2008; Cukrov et al., 2016; Brizzolara et al., 2017), and also in relation to the onset of low oxygen-related physiological disorders (Vandendriessche et al., 2013; Lumpkin et al., 2014). The metabolic responses including the activation of the fermentation pathway and the accumulation of related metabolites in apples stored under low oxygen/high carbon dioxide concentrations is highly variable depending on a number of factors, including the genotype, climatic conditions and agronomic practices, pre-treatments before storage, and CA protocols (Tonutti, 2015; Zanella and Sturz, 2015; Boeckx et al., 2019). “Granny Smith” and “Red Delicious” apples have different ACPs and accumulation patterns of ethanol, acetaldehyde, and lactate under static and DCA conditions; “Red Delicious” accumulates fermentative metabolites at higher oxygen concentrations (0.9 kPa) than “Granny Smith,” highlighting significant genotype differences (Brizzolara et al., 2017).

While accumulation of lactate is negligible during fermentative metabolism in CA-stored apples (Saquet and Streif, 2008), the transient increase of acetaldehyde and ethanol contents characterizes the early responses of apple fruits to oxygen concentrations below the specific ACP for a given cultivar. Interestingly, after an increase of fermentation metabolites, their concentration decreases after prolonged exposure to low oxygen stress (Cukrov et al., 2016; Brizzolara et al., 2017; Boeckx et al., 2019). The physiological mechanism responsible for this behavior remains to be elucidated.

The reduction of acetaldehyde to ethanol catalyzed by ADH is a key biochemical step in plant responses to hypoxic conditions. Specific *ADH* family members can be identified as core-responsive genes to hypoxia in apples. In fact, *ADH* genes are extremely reactive to changes in oxygen concentrations showing fine-tuned regulated expression of different members belonging to this multigene family (Cukrov et al., 2016; Brizzolara et al., 2019). However, the regulatory mechanisms of fermentative metabolism in fruits in response to low oxygen concentrations are complex. Based on kinetic modeling of responses of apple slices to low oxygen concentrations, two mechanisms were suggested by Boeckx et al. (2019): (1) the molecular control of the transcript and protein levels of PDC, ADH, and LDH and (2) the metabolic control of these enzymes by changing cytosolic pH set-points, cofactors, and substrate levels. These authors suggest that the fermentative metabolism is highly dependent on factors that modulate reaction rates, with both molecular and metabolic control systems being activated when the overall metabolic rate is fast.

Mannose and melibiose contents increased under low oxygen conditions. (Hatoum et al., 2014; Brizzolara et al., 2017). Together with other sugar alcohols, melibiose plays an osmoprotection role and it is involved in stress responses, accumulating in stress-tolerant species, and is a stress markers in plants (Hill et al., 2013). Increased sugar alcohols have been observed in different fruits kept at low oxygen concentrations (Pedreschi et al., 2009). Given their antioxidant potential, such compounds may act directly as protectant molecules, or in complementing carbon storage reserves under sugar shortages (Moing, 2000).

### Organic Acid Metabolism

A general decrease of organic acid concentrations in plant tissues may be a survival strategy aimed at reducing the flux toward nitrogen metabolism to keep on producing substrates for the glycolysis (Geigenberger, 2003; Galili, 2011; Ampofo-Asiama et al., 2014). Organic acids are key elements of the adaptive response of fruit tissue to low oxygen stress, although the overall pattern of organic acids in fruits stored under low oxygen and high carbon dioxide concentrations has still to be defined in detail. Succinate, a key metabolite in hypoxic responses, increases under low oxygen stresses in model systems (Bailey-Serres et al., 2012). Succinate also accumulates in apples and pears under high carbon dioxide concentrations (Vandendriessche et al., 2013; Bang et al., 2019), and this is associated with the inhibition of succinic dehydrogenase activity (Gonzalez-Meler et al., 1996). Accumulation of succinate has been correlated with the development of carbon dioxide-related physiological disorders (Pedreschi et al., 2008), although succinate can accumulate in the presence of the antioxidant diphenyamine, which prevents carbon dioxide injury (Fernandez-Trujillo et al., 1999). The effects of low oxygen concentrations in fruit tissues seem to be slightly different from those detected in rice and *Arabidopsis.* Lower levels of succinate have been detected in “Granny Smith” fruits kept at 0.4 kPa compared with those maintained at 0.8 kPa oxygen (Brizzolara et al., 2019), while “Red Delicious” fruit had higher succinate contents under static conditions at 0.9 kPa oxygen compared with 0.2–0.55 kPa oxygen (Brizzolara et al., 2017). One hypothesis is that GABA is catabolized to succinic semialdehyde (SSA) and then to succinate, with the latter reaction being catalyzed by SSA dehydrogenase (SSADH) in air-stored fruit. Under hypoxia, SSADH activity is likely reduced by increases in reducing potential and adenylate energy charge, thus resulting in the increase of GABA and the decrease of succinate. The effects of low oxygen/high carbon dioxide concentrations in fruits in relation to succinate metabolism need to be further elucidated.

The fate of malate is also affected by low oxygen concentrations. In general, the lower the oxygen concentration during storage, the greater the malic acid contents are maintained (Hatoum et al., 2016). Hypoxia induces aerobic respiration processes and affects specific TCA enzymes (Ampofo-Asiama et al., 2014). Normally, a decrease in malate content occurs in pome fruits after prolonged storage under hypoxia, which can be attributed to advanced ripening, and higher malate contents in the inner cortex compared with the outer cortex is probably the result of the lower respiration rate of this tissue due to reduced oxygen availability (Hatoum et al., 2016).

Fumarate contents increase under CA conditions, especially with higher carbon dioxide concentrations (Vandendriessche et al., 2013; Hatoum et al., 2016), perhaps as a result of down-regulation of fumarase, which catalyzes the hydration of fumarate to malate (Pedreschi et al., 2007). CA storage also may contribute to higher fumarate contents by promoting the conversion of oxaloacetate from phosphoenolpyruvate through the reversal of TCA reactions (Pedreschi et al., 2009).

In contrast, citrate contents in fruits are stable during air storage, but decline in CAs (Flaherty et al., 2018a). Citrate accumulation could result from the efflux of the vacuolar reserves, where most citrate is stored (Etienne et al., 2013). Citrate contents are also linked to glutamate fate since this latter is formed from 2-oxoglutarate (2-OG) and ammonia through the activity of cytosolic glutamate dehydrogenase, while 2-OG is produced from stored citrate (Sweetlove et al., 2010).

### Amino Acid Metabolism

In addition to the activation of the fermentative pathways to sustain glycolysis in the absence of mitochondrial respiration, nitrogen metabolism is profoundly affected by oxygen deprivation.

Amino acids linked to the TCA cycle are an integral component of respiratory metabolism and changes in their contents represent one of the main responses of plants to oxygen stress. Tolerant plants (e.g. rice) generally accumulate amino acids such as Ala, Ser, and Gly when subjected to low oxygen stress (Shingaki-Wells et al., 2011). The accumulation of Ala and GABA appears to be common responses of plants to hypoxia (Narsai et al., 2011). It has been hypothesized that Ala formation is important for removal of accumulated pyruvate under oxygen deficiency, thus indirectly contributing to pH homeostasis by competing with lactate dehydrogenase (Mustroph et al., 2014).

The contents of many different amino acids, such as Ala, Asp, GABA, Pro, Ser, and Thr in apples are modulated by oxygen concentrations during CA storage, with pyruvate-derived compounds and the expression of related metabolic genes affected differently by low oxygen concentrations (Cukrov et al., 2016).

Ala, which has been found to be a main hypoxia-related metabolite accumulating in several plant species (Bailey-Serres et al., 2012; Diab and Limami, 2016) including apples (Vandendriessche et al., 2013; Hatoum et al., 2014; Cukrov et al., 2016; Brizzolara et al., 2017), derives from pyruvate transamination *via* alanine aminotransferase (AlaAT) activity coupled to two different possible reactions: the production of 2-OG from glutamate or the generation of SSA from GABA (Hyun et al., 2013). The GABA shunt is less efficient at generating 2-OG and, thus, it is possible that alanine is derived from Glu under low oxygen concentrations, and that plant cells use it as storage form of pyruvate. Hatoum et al. (2016) reported increases of alanine contents in “Braeburn” apples after 8 months of CA storage. In “Granny Smith” apples Ala content increases very rapidly after low oxygen stress is imposed but, differently from ethanol, a less marked effect of oxygen concentration is present (Cukrov et al., 2016). *AlaAT* gene expression is highly induced by hypoxic stress in apple cortex and a rapid re-adjustment of transcription occurs in relation to oxygen concentrations applied under DCA conditions (Brizzolara et al., 2019).

GABA appears to play a key role in energy metabolism and defence against different abiotic stresses in fruits (Shang et al., 2011; Yang et al., 2011; Wang et al., 2016). GABA represents a key compound in the interface between C and N metabolism under energetically demanding stresses possessing a pivotal role in stress responses, due to its double role of acting directly as protectant compound and functioning as signalling molecule used by plant tissue to tune stress responses (Michaeli and Fromm, 2015; Takayama and Ezura, 2015; Diab and Limami, 2016). GABA synthesis is enhanced when the cytosolic pH decreases (Hyun et al., 2013), hence its production could also be related to the fate of the organic acids in the cell, possibly being a tissue- or treatment-specific event also uncoupled from the response to low oxygen concentrations itself. The accumulation of GABA is highly dependent on the oxygen concentrations (Cukrov et al., 2016) and enhanced concentration of GABA are considered as a marker of hypoxia in apples and pears (Pedreschi et al., 2009; Cukrov et al., 2016). In addition, GABA catabolism is highly reactive and sensitive to oxygen and carbon dioxide changes. Decreases of GABA content have been detected in apple cortex tissues within 24 h of oxygen shift from 0.4 to 0.8 kPa (Brizzolara et al., 2019) and two apple GABA transaminase (GABA-T) genes are rapidly up-regulated in apples moved from CA to normoxic conditions (Trobacher et al., 2013). GABA contents have been also reported to increase in strawberries and tomatoes during postharvest treatment with 20 kPa CO_2_ (Deewatthanawong et al., 2010a; Deewatthanawong et al., 2010b).

Overall, Ala and GABA production are important adaptive process allowing carbon and nitrogen storage, as well as acting as osmoprotectants under stress conditions, and balancing a rapid decrease in carbohydrates. Ala production under hypoxic stress represents an adaptive carbon allocation strategy that maintains the glycolytic flux, limits pyruvate accumulation and, at the same time, maintains carbon resources in the cell (Rocha et al., 2010). In fact, the fermentative pathway and ethanol production result in NAD+ regenration thereby allowing glycolysis to proceed to sustain ATP production, but the pathway drains carbon for the production of metabolically useless dead-end products (Limami, 2014). In addition, the interconversion of other amino acids normally takes place at cellular level acting as a tool for adapting cell metabolism/homeostasis to different environments. Therefore, hypoxia affects the fate also of other compounds belonging to this chemical class. Among them, Glu, Pro, as well as Ile, Thr, and Ser production/accumulation increase, while Asp and Asn contents showed a decrease in fruits stored under low oxygen concentrations (Lee et al., 2012; Hatoum et al., 2014; Bekele et al., 2016; Cukrov et al., 2016; Brikis et al., 2018). These results are mainly in agreement with the role of these amino acids as precursors for Ala and GABA production (Oliveira and Sodek, 2013).

### Lipid Metabolism

Lipid metabolism in fruits under CA conditions is not well studied. Lipid biosynthesis is characterized by several pathways involving enzymes that require oxygen for their activity, such as the ones involved in the biogenesis of sterols and the desaturation of fatty acids (Harwood, 1988; Ohlrogge and Browse, 1995), and which may be affected by hypoxia. Indeed, lipid biosynthesis and desaturation have been since long time recognized to decrease in plants subjected to low oxygen concentrations (Brown and Beevers, 1987).

An early study postulated that phospholipids accumulated more slowly in apples stored in CA than in air (Bartley, 1986), while Brackmann et al. (1993) found lower peel fatty acid content in CA- than air-stored apples. CA storage affected the biosynthetic mechanisms of these compounds, especially linoleic acid. How different oxygen concentrations exert their effect on such pathways is still unclear. Production of unsaturated fatty acids, such as linoleic acid, requires oxygen and this partly explains their lower content under CA conditions. However, lipid biosynthesis could also be reduced because CA conditions inhibit respiration and general metabolism, thereby reducing the available energy equivalents, such as NADPH, that are needed for fatty acid synthesis (Ho et al., 2013). Delaying CA storage of apples results in higher contents of fatty acids, and polar lipids, including phospholipids after storage (Saquet et al., 2003). In addition to the energy issue, another possible cause of the observed decrease of lipids under low oxygen conditions involves ethylene production and action, which are lower after CA storage: in apples a pre-storage treatment with the inhibitor of ethylene biosynthesis, aminoethoxyvinylglycine, decreased ATP and fatty acid levels under storage in normoxia (Halder-Doll and Bangerth, 1987).

Higher PCs and phosphatidylethanolamines (PEs) and lower arachidonic acid contents, the latter a product of PC and PE metabolism, have been observed in apple cortex one day after a partial re-oxygenation of the fruit when compared with apples stored constantly at low oxygen regimes (Brizzolara et al., 2019). This study reported that after oxygen re-supply the up-regulation of two genes encoding phospholipase A2 and the down-regulation of two phospholipases (C2 and D delta) occurred, indicating that phospholipid metabolism is highly reactive to slight changes in oxygen concentrations within the storage rooms. These results also pointed out that a transient burst of phospholipid metabolism could play an important role in the early responses of apple cortex cells subjected to oxygen re-supply, as well as in the generation of regulatory signals. Phosphoglycerides are main components of cell membranes, and mitochondria membranes are particularly rich in PC and PE (Harwood, 1987). Lipid metabolism in plants is altered under stress conditions: Xie et al. (2015) demonstrated that in hypoxic *Arabidopsis,* rosettes are characterized by increases in unsaturated glycerolipid species, PA, as well as oxidized membrane lipids, and decreases of phosphatidylglycerols (PGs), PCs, and PEs.

## Ethylene Effects on Primary Metabolism of Harvested Fruits

The role of ethylene has long been recognized as a critical factor regulating ripening and senescence, and subsequently the storage life, of an array of horticultural commodities (Abeles et al., 1992; Saltveit, 1999; Tucker et al., 2017). Ethylene can affect the quality of these crops either when produced endogenously, or exogenously either applied to positively affect a biological factor, or as a contaminant. Low temperature and CA/MA affect product quality beneficially by decreasing metabolic activity in part by reducing ethylene production and action.

Fruits have traditionally been separated into two distinct groups, climacteric and non-climacteric. In climacteric fruits (and fruit vegetables) such as apple, avocado, banana, pear, and tomato, ripening is associated with an increase in respiratory activity that is associated with autocatalytic production of ethylene. Climacteric fruit, if harvested when mature, continue to ripen. In contrast, the respiration rates of non-climacteric fruits such as citrus, strawberry, and grape, decline during ripening with no autocatalytic ethylene production, and coordinated ripening changes do not take place after harvest. However, both climacteric and non-climacteric fruits respond to ethylene, but in very distinct ways that can be used as diagnostic tools (Wills and Golding, 2016). When progressively higher ethylene concentrations are applied to climacteric fruits, the size of the respiratory peak remains similar, but the timing of the respiratory maximum (and ripening) is accelerated even after treatment stops. In a non-climacteric fruit, respiration rates are proportionally greater as the ethylene concentration is increased but timing of the peak is not affected, and respiration decreases as soon as ethylene is removed. The rate of ripening is not necessarily a function of ripening type: non-climacteric fruits such as sweet cherries and strawberry can ripen rapidly, while citrus ripen slowly; climacteric fruits such as avocados and pears ripen rapidly, while certain apple cultivars ripen slowly.

However, a strict distinction between climacteric and non-climacteric fruits is less clear than originally thought (Paul et al., 2012). Fruit types that have both ripening patterns include melon and plums (Abdi et al., 1997; Fernandez-Trujillo et al., 2008; Farcuh et al., 2019). It has long been thought that the ripening of climacteric fruits is regulated by ethylene while that of non-climacteric fruits is regulated by abscisic acid (ABA) (Jia et al., 2011; Jia et al., 2013). However, it is clear that ethylene and ABA, as well as other hormones (e.g. auxins), are involved in ripening of both types of fruits (Manganaris et al., 2011; Li et al., 2019). Climacteric and non-climacteric types also share many aspects of ethylene perception and signaling and interestingly, the EThylene Receptor (*ETR*) gene is more abundant in climacteric fruit than non-climacteric fruits, and ETR accumulates earlier in the latter (Chen et al., 2018).

The involvement of ethylene in primary metabolism has been explored in many ways that include investigations into metabolism of normally ripening fruits, use of mutants or transgenic approaches, ethylene treatment of fruits with ethylene [directly as a gas or by use of ethylene releasing agents such as 2-chloroethylphosphonic acid (ethephon)], and ethylene analogues such as propylene. Ethylene antagonists such as silver and 1-MCP also have been used; of the two, 1-MCP has been especially useful for researchers due to its non-toxic nature, gaseous form at physiological temperatures, and its effectiveness at low concentrations (Sisler and Serek, 2003; Watkins, 2015). Although ethylene concentrations around fruits can also be lowered by methods such as ventilation, avoidance, and absorbers and oxidizers (Martinez-Romero et al., 2007), there is little information about their effects on primary metabolism.

In general, application of ethylene to climacteric fruits accelerates the rate of ripening (Saltveit, 1999), while 1-MCP decreases the rate of ripening, and in some cases can totally inhibit it (Watkins, 2006; Watkins, 2015). However, 1-MCP induces variable responses in different climacteric fruit species and even in cultivars of the same species (Watkins, 2015). Ethylene biosynthetic and signal transduction pathways of apples and peaches are differentially affected by 1-MCP action (Dal Cin et al., 2006). 1-MCP can also affect various ripening/senescence processes of non-climacteric fruits (Huber, 2008; Li D. et al., 2016; Kafkaletou et al., 2019).

Non-targeted genomic, proteomic, and metabolomic approaches have revealed a number of changes in primary metabolism in response to ripening, exogenous ethylene, and ethylene inhibitors (Giovannoni et al., 2017; Li et al., 2017; Zhao X. et al., 2019). In nectarines, about 30% of the transcriptome corresponded to genes involved in primary metabolism and response processes related to ethylene, auxin, and other hormones (Ziliotto et al., 2008). In 1-MCP-treated fruits, altered transcript accumulation was detected for some genes with roles in ripening-related events including sugar metabolism. Changes in proteins of apples during ripening revealed enzymes involved in gluconeogenesis, C-compounds and carbohydrate metabolism, electron transport/energy production, and malic acid metabolism. Proteins involved in several multiple metabolic pathways, including glycolysis and the pentose-phosphate pathway were down-regulated, especially during the climacteric burst in respiration and during the senescent stage (Shi et al., 2014). In peaches, 34% of differential protein expression in response to 1-MCP and ethephon were associated with pathways involved in energy and general metabolism (Zhang et al., 2012). In CA-stored “Empire” apples, most carbohydrates and organic acids were not appreciably affected by 1-MCP treatment, but levels of sorbitol and some amino acids were elevated toward the end of storage in treated fruit (Lee et al., 2012).

### Carbohydrate Metabolism

Most research on the effects of ethylene on starch metabolism in fruit has been performed on bananas, and to a lesser extent on apples and kiwifruits. Starch degradation and sucrose synthesis are both developmentally regulated at transcriptional level (Janssen et al., 2008; Xiao et al., 2018; Zhang et al., 2018; Yan et al., 2019). Examples include kiwifruits, where analysis of the regulatory effects of differentially expressed genes identified a zinc finger TF, DNA BINDING WITH ONE FINGER (AdDof3), which showed significant transactivation on the *AdBAM3L* (β-amylase) promoter (Zhang et al., 2018). In apples, ethylene biosynthesis is inhibited in the antisense-suppressed MADS8as-9 line; ethylene application to this line partially stimulated ripening, with starch degradation and other late ripening processes not being complete (Ireland et al., 2014).

Starch hydrolysis in bananas was enhanced by treatment with propylene or ethylene (Saraiva et al., 2018). Starch hydrolysis in apples can be dependent or independent of ethylene concentration, whether exogenous or endogenous (Thammawong and Arakawa, 2007; Doerflinger et al., 2015a). Postharvest 1-MCP effects on starch hydrolysis in apples also can be cultivar-dependent (Fan et al., 1999; Neuwald et al., 2010; Thammawong and Arakawa, 2010; Doerflinger et al., 2015b). In bananas, 1-MCP delays hydrolysis in a dose- and cultivar-dependent manner (Nascimento et al., 1997; Mainardi et al., 2006). Variation of responses to ethylene in different fruit types and cultivars may be explained by differences in sensitivity (Johnston et al., 2009); initiation of starch hydrolysis is ethylene sensitive and therefore even a very small increase in ethylene concentration can trigger the onset of starch hydrolysis. However, once initiated its hydrolysis is relatively independent of ethylene concentrations within the fruit tissue. Also, relationships between ethylene and starch metabolism are not straightforward, as starch degradation in bananas, for example, is coordinated with that of cell wall softening (Shiga et al., 2011).

Simultaneous synthesis and degradation of sucrose during postharvest storage of fruits has been well described (Duque et al., 1999; Zhu et al., 2013). A link between ethylene and sucrose metabolism is suggested by increased gene transcript and enzymatic activity of SPS by exogenous ethylene treatment or during postharvest ripening (Duque et al., 1999; Choudhury et al., 2008; Lombardo et al., 2011). Sucrose treatment of tomato fruits advanced ripening, and increased expression of genes involved in both sugar biosynthesis and degradation (Li L. et al., 2016). The *ETR* transcripts accumulated earlier in non-climacteric than in climacteric fruits, and this expression coincided with the onset of sugar accumulation (Chen et al., 2018). Investigations with “Micro-Tom” tomatoes and five hormone mutants (namely *dpy*, *not*, *dgt*, *epi*, and *pro*) indicated that ethylene plays an important role in regulation of sugar accumulation (Li et al., 2019).

A feature of 1-MCP-treated apples is the low sucrose contents compared with the controls (Defilippi et al., 2004; Bekele et al., 2015). Bekele et al. (2015) suggested that low sucrose was due to temperature mediated activation of sucrose degrading enzymes and suppression of SPS activity by 1-MCP treatment, whereas in untreated fruit, the breakdown of sucrose did not result in accumulation of glucose due to its utilization to sustain a high respiration rates. However, 1-MCP treatment increased *SuSy* expression associated with the sucrose–sucrose cycle in “Royal Gala” apples (Storch et al., 2017). It was suggested that in the absence of ethylene, the fruit resumes a set of metabolic activities that were suppressed during ripening in the presence of ethylene. Sucrose contents are also lower in fruit from trees with downregulated 1-aminocyclopropane-1-carboxylic acid (ACC)-synthase or ACC-oxidase enzyme activity, but contents could be restored to similar levels as wild type after exposure to ethylene (Defilippi et al., 2004).

Although grapes as non-climacteric fruits are thought to be ethylene independent, 1-MCP treatment resulted in lower sucrose accumulation in berries than in untreated fruit (Chervin et al., 2006). Decreased sucrose accumulation was associated with a down-regulation of two sucrose transporters (*SUC11* and *SUC12*), whose expression is triggered at veraison when grape berries start to accumulate sugars. In citrus, 1-MCP repressed genes involved in starch synthesis and degradation and increased the expression levels of SuSy genes (Estables-Ortiz et al., 2016). In grapes, and another non-climacteric fruit, strawberry, it has been proposed that sucrose functions as a signal that acts upstream of the ABA signaling pathway, thus playing an important role in the regulation of fruit ripening (Jia et al., 2013; Jia et al., 2017).

A mutant series of Japanese plums exhibits three distinct ripening patterns: climacteric, suppressed-climacteric, and non-climacteric (Minas et al., 2015). “Santa Rosa,” which is climacteric, and its bud-sport mutant “Sweet Miriam,” which is non-climacteric, have been used to investigate sugar metabolism. At the ripe stage, non-climacteric fruits accumulate higher sorbitol than that of climacteric fruits (Kim et al., 2015; Farcuh et al., 2017; Farcuh et al., 2018). Higher sorbitol contents in “Sweet Miriam” were associated with decreased activity and expression of NAD^+^-dependent sorbitol dehydrogenase and sorbitol oxidase and increased sorbitol-6-phosphate dehydrogenase activity, as well as increased sucrose catabolism (Kim et al., 2015). Enhanced sorbitol synthesis and lower sucrose, glucose, and fructose contents were also found during on-tree ripening of the non-climacteric “Sweet Miriam” than in the climacteric “Santa Rosa” (Farcuh et al., 2017); contents of the minor sugars galactinol, raffinose, *myo*-inositol, and trehalose also increased in “Sweet Miriam,” while that of galactose was higher in “Santa Rosa.” The effects of ethylene on sugar metabolism were studied using propylene and 1-MCP (Farcuh et al., 2018). Ethylene increased biosynthesis of sucrose while decreasing that of sorbitol. Decreased and increased sucrose and fructose accumulation may result from sorbitol and sucrose catabolism in climacteric and non-climacteric fruits, respectively. Ethylene resulted in galactose accumulation in “Santa Rosa”; galactose has been also reported to stimulate ethylene production in tomato fruits (Kim et al., 1987).

### Organic Acids

A general pattern of organic acid contents of fruits during development, is an initial accumulation of acids followed by a decrease of contents either by increasing fruit mass or metabolism as a function of synthesis, degradation, and compartmentalization. The acids are sequestered in the vacuoles and released to provide substrate for increased respiration during ripening of climacteric fruits, meet higher energy demand in non-climacteric fruits, and produce hexoses by gluconeogenesis (Beruter, 2004; Sweetman et al., 2009). Influx and efflux of malic acid from the vacuole may vary over time (Walker et al., 2015; Famiani et al., 2016b). In peaches, contributions of malic and citric acids to metabolism are negligible, and it is likely that gluconeogenesis occurs during ripening (Famiani et al., 2016b). In ripening of grapes, a non-climacteric fruit, decreased malic acid contents were associated with metabolism, but to a lesser extent compared with sugars (Famiani et al., 2016a). Overall, there is a diversity of responses of different fruits to ethylene and the contribution of stored organic acids to metabolism is not well understood. Organic acid metabolism is not directly linked to respiratory and climacteric characteristics of the fruit, however, interactions between organic acid metabolism and hormone signaling provide useful insights for future research (Batista-Silva et al., 2018).

Detailed studies exploiting 1-MCP to investigate organic acid metabolism are recent. In apples, 1-MCP slowed the decreases of malic and citric acid contents during ripening at 20°C by regulating organic acid metabolism (Liu et al., 2016). In addition, up-regulation of *Md PEPC* and *MdcyMDH* expression, higher PEPC and cyNAD-MDH activities, and the decreased malate degradation *via* limiting *MdPEPCK* expression with lower activity of PEPCK were reported. 1-MCP treatment up-regulated acid transport genes, including *MdVHA-A*, *MdVHP*, and *Ma1*, resulting in higher malic acid contents in the vacuole (Liu et al., 2016). In Asian pears, decreasing malic acid contents during ripening were associated with down-regulation of genes associated with malic acid metabolism, lower cyNAD-MDH, and higher cyNADP-MDH activity (Wang et al., 2018). 1-MCP-treated fruits had higher malic acid contents, upregulated gene expression, higher NAD-dependent malate dehydrogenase activity, and lower NADP-ME-dependent malate dehydrogenase activity.

### Amino Acids and Lipids

Amino acid contents typically decrease during ripening and storage, often associated with secondary pathways that produce aroma volatiles, particularly esters, because of the close linkages with ethylene production during ripening (Giovannoni et al., 2017). 1-MCP treatments of fruits can maintain higher amino acid contents (Lee et al., 2012; Zhang et al., 2014; Bekele et al., 2015) that could result from a reduced utilization in metabolic processes. Amino acids may also be involved in fruit responses to stress and development of physiological disorders associated with 1-MCP treatment through their role as substrate for compounds such as GABA (Flaherty et al., 2018b). They are also directly linked to many secondary metabolic processes, most notably production of aroma volatiles, several groups of which are related to amino acid and fatty acid metabolism.

Loss of membrane function with changes in membrane lipids, particularly their degree of unsaturation, is a feature of ripening and senescence (Marangoni et al., 1996). These changes lead to altered membrane properties and result in defects such as ion leakage and loss of cellular compartmentalization, and therefore represent an obvious consequence of ethylene production. The literature on these changes outside of the effects of imposed postharvest treatments such as low temperature and CA/MA is limited. While 1-MCP has been used to investigate the effects of ethylene on lipids it is not surprising that the results typically show slowing down of changes associated with ripening and senescence. In fruits such as kiwifruit, pear, pitaya, and tomato, 1-MCP inhibited lipid associated changes such as increases of membrane permeability, lipid peroxidation, the decrease of unsaturated fatty acids and *PLD* gene expression (Dek et al., 2018; Huang et al., 2019; Tao et al., 2019; Xu et al., 2019).

## Final Remarks and Future Perspectives

Storage protocols are based on the application of controlled stresses or treatments aimed at delaying genetically programmed fruit ripening and the onset of senescence by affecting primary metabolism.

This review highlights a number of issues specifically dealing with the effects of three main post-harvest physical and chemical factors on the primary metabolic processes of stored fruits. The effects of these protocols depend on a number of factors including the intensity and duration of the applied protocol, the fruit type and cultivar, the maturity and ripening stage at harvest, as well as pre-harvest conditions. Considering the impact of pre-harvest factors, the information available concerns solely the effects of specific treatments and protocols (e.g. spraying with chemicals, controlling crop load, fertilization, water management) on the fruit responses in terms of shelf-life, technological parameters and the incidence of decay and disorders, with no specific information regarding primary metabolic pathways.

An extensive literature on ethylene and our understanding of its involvement in ripening of both non-climacteric and climacteric fruits has been developed. Transgenic and 1-MCP treatments have become powerful tools to investigate the role of ethylene on primary metabolism. Segregation of fruits based on ripening patterns are less strict than previously assumed and cross talk among hormones other than ethylene, has been identified. The interactions between ethylene signaling and sugars also is an especially active area, while there is a paucity of information about organic acids, amino acids and lipids. Studies concerning postharvest treatments such as low temperature and the effects of CA/MA have centered more on injuries associated with these treatments rather than on the effect of these treatments on non-injurious metabolism. High variability of responses associated with different genotypes to an array of postharvest treatments, often used in an integrated manner, renders difficult to identify primary metabolic changes occurring in fruit tissues in contrast to model systems.

The increasing availability of genome sequencing of different genotypes, and the development of “omics” techniques are providing tools to overcome these limitations, and thereby better understand and clarify the fundamental mechanism regulating the postharvest metabolic responses. These tools are also providing opportunities to exploit findings that are, so far, mainly based on model systems. This is the case of low oxygen and high carbon dioxide postharvest stress physiology studies. Recent reports describe the role of specific transcription factors (TFs) such as ethylene-responsive factors (ERFs), in synergy with WRKY and MYB elements, in controlling the expression of *PDC* gene promoter in persimmons under high carbon dioxide/hypoxia (Zhu et al., 2018; Zhu et al., 2019). Specific and multiple TFs of different clades/classes and a TF regulatory network are involved in the responses to such storage conditions that induce marked changes of primary metabolism gene expression. *ADH* and *PDC*, belonging to the core responsive genes of plants to low oxygen conditions (Mustroph et al., 2009), are controlled by ERFs in *Arabidopsis*, through a fine-tuned mechanism (N-end rule pathway, NERP) of oxygen sensing (Gibbs et al., 2011; Licausi et al., 2011). ADH and PDC enzymes are involved in primary (fermentative) metabolism and play a major role in the determination of fruit flavor and aroma. In apples, a specific ERF protein (MdRAP2.12) has been shown to differentially accumulate at different oxygen concentrations, suggesting that the oxygen-sensing mechanisms described in *Arabidopsis* are also present in apple fruit (Cukrov et al., 2016). The variable responses in terms of the primary metabolism compound (pyruvate, alanine, ethanol) accumulation observed in different apple cultivars under hypoxic conditions might be the result of the selective activation and specific organization of ERF-based sensing mechanisms (Brizzolara et al., 2017).

An additional field of investigation involves the tolerance of fruits to cold stress occurring during storage. Cold-stored fruits re-direct their metabolism, starting from changes in gene expression, with different levels of tolerance to cold stress depending on the genetic background. A number of cold-responsive (COR) genes have been identified in vegetative tissues of model species, some of them involved in physiological and biochemical changes during the process of cold acclimation and tolerance. COR genes have been identified also in different fruit species such as peaches where their expression has been analyzed in relation to different behavior of CI sensitive and CI non-sensitive cultivars during refrigerated storage (Bustamante et al., 2016; Nilo-Poyanco et al., 2019). Selective changes in expression of genes related to energy and stress response, amino acid, carbohydrate, lipid, and specialized metabolism have been observed (Zhang et al., 2010; Pons et al., 2014; Pons et al., 2016) with C-repeat-binding factors (CBFs) apparently playing a key role in modulating the expression (Liang et al., 2013).

Elucidation of these regulatory mechanisms will widen our understanding on primary metabolic, and associated secondary, responses of fruit tissues of harvested fruits, and also help to optimize storage protocols with benefits in terms of reduced losses and improved consumer satisfaction.

## Author Contributions

All authors planned the structure of the review, contributed in writing the article, read and approved the submitted version.

## Conflict of Interest

The authors declare that the research was conducted in the absence of any commercial or financial relationships that could be construed as a potential conflict of interest.
